# *Dimerocostus strobilaceus* (Caña Agria) as an Emerging Reservoir of Bioactive Metabolites with Potential Antioxidant, Antimicrobial, Anticancer and Anti-Inflammatory Health Benefits

**DOI:** 10.3390/antiox14111298

**Published:** 2025-10-29

**Authors:** Elena Coyago-Cruz, Rebeca Gonzalez-Pastor, Gabriela Méndez, Jeico Usinia-Carranza, Juan A. Puente-Pineda, Johana Zúñiga-Miranda, Marco Cerna, Jorge Heredia-Moya

**Affiliations:** 1 Carrera de Ingeniería en Biotecnología, Universidad Politécnica Salesiana, Sede Quito, Campus El Girón, Av. 12 de Octubre N2422 y Wilson, Quito 170143, Ecuador; gmendez@ups.edu.ec (G.M.); mcerna@ups.edu.ec (M.C.); 2 Centro de Investigación Biomédica (CENBIO), Facultad de Ciencias de la Salud Eugenio Espejo, Universidad UTE, Quito 170527, Ecuadorjuan.puente@ute.edu.ec (J.A.P.-P.); jorgeh.heredia@ute.edu.ec (J.H.-M.)

**Keywords:** bioactive compounds, functional food, cell cultures, macrophages, tumour cell lines

## Abstract

The Amazon region is home to a wide variety of plant species that are of interest for their medicinal and nutritional properties. This study aimed to evaluate the physicochemical properties, bioactive compound profile, and antioxidant, antimicrobial, anticancer and anti-inflammatory activity of *Dimerocostus strobilaceus* leaves, stems, and seeds. Vitamin C, organic acids, carotenoids, and phenolic compounds were quantified using RPLC. Antioxidant activity was determined using ABTS and DPPH, while antimicrobial activity was assessed against pathogenic and multidrug-resistant bacteria. Anticancer activity was evaluated in tumour cell lines (HeLa, HCT116, HepG2, and THJ29T), and anti-inflammatory activity was examined in RAW 264.7 macrophages. The seeds stood out for their content of ferulic, caffeic and chlorogenic acids, while kaempferol and quercetin predominated in the leaves. The leaves exhibited greater antimicrobial efficacy and antiproliferative activity (IC_50_ < 0.2 mg/mL), albeit with limited selectivity. These findings suggest that ‘caña agria’ is a promising source of bioactive metabolites with biotechnological and therapeutic applications.

## 1. Introduction

The therapeutic use of plants dates to ancient civilisations such as Mesopotamia, Egypt, Greece, and the Islamic world. Sumerian tablets dating back over 5000 years document the medicinal use of species such as *Glycyrrhiza glabra* and *Papaver somniferum*. While the development of synthetic drugs has transformed modern medicine, plants remain a primary source of bioactive metabolites, with over 250,000 recognised species and considerable chemical potential [1].

In India, traditional medical systems such as Ayurveda, Siddha and Unani have used medicinal herbs to treat chronic diseases. The efficacy of these systems is attributed to the action of secondary metabolites. It has been validated in numerous scientific studies, which demonstrate radical-scavenging, anti-inflammatory, antibacterial/antifungal, antihypertensive and anticancer activities [2,3]. These compounds include flavonoids, alkaloids, terpenoids, quinones and phenolic compounds. They are known to modulate critical biochemical pathways such as inflammation modulation, cell apoptosis induction, tumour proliferation inhibition and antioxidant activity [4,5], making them promising candidates for the development of new drugs with greater specificity and lower toxicity [6].

Curcumin, quercetin and resveratrol have been shown to neutralise reactive oxygen species, prevent cell damage and modulate intracellular signalling pathways associated with cancer development [7]. Meanwhile, an increasing number of studies have reported the antimicrobial activity of plants against clinically relevant pathogens. A recent meta-analysis identified species such as *Lavandula* spp., *Plectranthus* spp. and *Lupinus jaimehintoniana* as being highly effective against *Escherichia coli* [8]. In Italy, 135 species were reported to be effective against Gram-positive bacteria, and 88 against Gram-negative bacteria [9]. Nevertheless, more comprehensive in vitro and in vivo experiments are essential to confirm these findings and develop safe and effective therapies [10].

Knowledge of the healing properties of plants has been passed down through the generations, and today, their importance to public health and biomedical research is widely recognised. The World Health Organisation reports that nearly 40% of the global population makes use of traditional medicinal practices [4]. The study of the phytochemistry of these species has led to the discovery of numerous drugs that are currently in clinical use, many of which are derived directly from secondary plant metabolites [11]. For example, *Andrographis paniculata* has demonstrated potent cytotoxic activity against colon and cervical cancer cell lines, with IC_50_ values comparable to those of doxorubicin [12]; *Annona muricata* has shown antiproliferative effects in preclinical models of breast cancer [13]; *Arum palaestinum*, which is traditionally used in the Middle East, has been confirmed as an inhibitor of tumour cell growth [14]; and *Catharanthus roseus*, which is a source of alkaloids such as vincristine and vinblastine, remains fundamental to chemotherapy protocols for leukaemia and other types of cancer [15].

Furthermore, traditional medicine plays a crucial role in megadiverse countries like Ecuador. Indigenous communities use over 120 species for therapeutic purposes, many of which have not yet been characterised scientifically. The country’s geographical and climatic diversity has led to the evolution of distinctive species with pharmacological properties, offering opportunities for bioprospecting new therapeutic agents [16]. One such species is *Dimerocostus strobilaceus* Kuntze, commonly known as ‘caña agria’, a perennial herbaceous plant that grows up to six metres in height. It is distributed throughout tropical regions, from Honduras to Bolivia [17,18]. This species has received little attention in relation to its therapeutic potential. Recent studies suggest that other species in the same genus may contain metabolites with significant biological activity, including antioxidant and anticancer properties. Natural compounds, such as alkaloids, terpenoids, and isothiocyanates, have been reported to modulate epigenetic pathways involved in cancer development by regulating gene expression, inducing apoptosis, and inhibiting tumour cell proliferation [19].

Against this backdrop, the present study aimed to evaluate the bioactive profile of *Dimerocostus strobilaceus* by quantifying its bioactive compounds and determining its antioxidant, antimicrobial, anticancer and anti-inflammatory properties. Studying this underutilised species will contribute to the promotion of Ecuadorian biodiversity and its use in developing natural therapeutic agents.

## 2. Materials and Methods

### 2.1. Plant Material and Physicochemical Analysis

**Figure 1:**
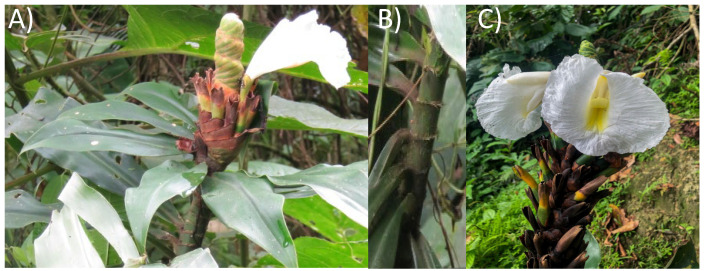
Photograph of *Dimerocostus strobilaceus* (Caña agria) leaves (**A**), stem (**B**) and seeds (**C**).

The study included the collection of leaves, stems, and seed samples of *Dimerocostus strobilaceus* from the Amazon rainforest in the province of Pastaza (1°44′21″ S, 77°29′1″ W) (Figure 1) during the rainy season in March. Samples were randomly selected the same location. Samples were taken from several different plants and mixed to create a composite sample. To ensure accurate botanical identification, the collected specimens were pressed and dried (Identification code: 4790, Herbario QUPS-Ecuador). Physicochemical properties of the samples were analysed, including pH, soluble solids (°Brix), total titratable acidity (%) was assessed by titration with sodium hydroxide 0.1 M [20], humidity (%), ash (%) and minerals content (Ca, Fe, K, Mg and Na) [21].

The pH was determined using a SevenMultiTM S47 pH meter (Mettler Toledo, Greifensee, Switzerland). Soluble solids were measured using a handheld refractometer. Total titratable acidity was assessed by titration. Moisture and ash content were determined gravimetrically. Moisture was measured at 70 °C using a Be20 oven (Memmert GmbH Co. KG, Schwabach, Germany). At the same time, ash content was obtained after incineration at 550 °C in a Thermolyne muffle (Thermo Fisher Scientific, Waltham, MA, USA). Minerals were quantified using an atomic absorption spectrophotometer with a Varian SpectrAA-55 instrument (Agilent Technologies, Santa Clara, CA, USA). Determination was performed at a specific wavelength, employing calibration curves derived from standard reference solutions. Mineral profile were measured in milligrams per 100 g of dry weight (mg/100 g DW) [21].

### 2.2. Plant Extract

The different parts of *D. strobilaceus* under study were deep-frozen at −80 °C before lyophilisation using a Christ Alpha 1–4 LDplus (GmbH, Osterode am Harz, Germany). Research has shown that this method contributes to the preservation of bioactive compounds [22,23]. Once dried, the material was placed in amber glass bottles filled with nitrogen and kept frozen until further analysis of bioactive compounds and biological activities.

### 2.3. Phytochemical Screening

The extract was obtained by suspending 20 mg of lyophilised powder in 1000 µL of deionised water (1:10 weight/volume ratio). This was then homogenised and sonicated for 3 min in an FS60 ultrasonic bath (Fisher Scientific Inc., Waltham, MA, USA). The supernatant was collected, after which the solid residue was re-extracted twice more using 500 µL of water each time. Successive reactions were then carried out with different reagents on the recovered extract to verify the presence of alkaloids, acetogenins, anthraquinones, flavonoids, phenols, saponins, steroids, tannins and terpenoids, in accordance with the methodology outlined by León-Fernández et al. [24].

### 2.4. Bioactive Compounds

#### 2.4.1. Vitamin C Identification

The extract was carried out in triplicate by suspending 40 mg of freeze-dried material with 200 μL of a 0.2% *DL*-homocysteine solution and 1.2 mL of 3% metaphosphoric acid. This was then homogenised and subsequently agitated on a Fisher Scientific FS60 ultrasonicator (Fisher Scientific, Hampton, NH, USA) for a period of one minute. Following homogenisation, deionized water was added to bring the total volume to 2 mL.

Phase preparation was achieved by centrifugation at 13,171× *g* for 5 min at 4 °C in a centrifuge Eppendorf 5430 (Eppendorf AG, Hamburg, Germany). Then, the supernatant was filtered through a 0.45 µm PVDF filter and transferred into a 2 mL autosampler vial for subsequent analysis. Chromatography determination was performed using an Agilent 1200 series rapid resolution liquid chromatograph (RRLC) (Agilent Technologies, Santa Clara, CA, USA) equipped with a DAD-UV-Vis detector at 244 nm. Separation was achieved on a Zorbax Eclipse column, XDB-C18, 80 Å pore size (4.6 × 50 mm, 1.8 µm particle size, operating pressure 600 bar) (Agilent Technologies, Santa Clara, CA, USA) maintained at 30 °C. A mobile phase consisting of 1.5% monobasic potassium phosphate and 1.8% *n*-acetyl-*n*,*n*,*n*-trimethylammonium bromide in a 90:10 (*v*/*v*) ratio was used for the chromatographic separation, at a flow rate of 1 mL/min.

Each sample was injected twice (20 µL injection volume) and monitored using Open Lab ChemStation software (version 2.15.26). Vitamin C identification was based on comparison of retention time, spectral characteristics at 244 nm, and the use of an internal standard. A standard solution of *L*-(*+*)-ascorbic acid (1 mg/mL) was employed for calibration, and the results were reported as milligrams per 100 g of dry weight (mg/100 g DW) [25].

#### 2.4.2. Organic Acid Identification

A 40 mg freeze-dried sample was weighed and suspended in 1.5 mL of 0.02 N sulfuric acid supplemented with 0.05% metaphosphoric acid and 0.02% *DL*-homocysteine. The mixture was thoroughly vortexed and subsequently subjected to ultrasonic bath treatment for 3 min to enhance the extraction. Deionised water was added to bring the total volume to 2 mL. Phase separation was achieved by centrifugation of the suspension at 13,171× *g* at 4 °C for 5 min. Subsequently, the supernatant was filtered using a 0.45 µm PVDF filter and collected into a 2 mL autosampler vial for chromatographic analysis.

Analysis was conducted using an Agilent 1200 series RRLC system coupled to a diode array detector DAD-UV-Vis (Agilent Technologies, Santa Clara, CA, USA) set at 210 nm. Chromatographic separation was performed on a YMC-Triart C18 column (150 × 4.6 mm, 3 12 nm, 400 bar) (YMC Europe GmbH, Dinslaken, Germany). The mobile phase consisted of 0.027% sulfuric acid at a flow rate of 1 mL/min, with a total analysis time of 30 min and an injection volume of 20 µL per sample. All injections were performed in duplicate.

Organic acids were identified through comparison of their retention times, UV-Vis spectral profiles with reference standards at 210 nm, and the use of internal standards. Concentration was determined by constructing individual calibration curves for *L*-(*+*)-tartaric, citric, and malic acids standard solutions (100 mg/mL). Results were expressed as miligrams per 100 g of dry weight (mg/100 g DW), and total organic acid content was calculated as the sum of the individual concentrations of the identified acids [25].

#### 2.4.3. Carotenoids Identification

For sample preparation, triplicated aliquots of 20 mg of freeze-dried material were weighed and treated with 250 µL of methanol, 500 µL of dichloromethane and 250 µL of acetone. The mixtures were homogenised using vortex agitation, followed by ultrasonication for 2 min. After centrifugation at 13,171× *g* and 4 °C for 5 min, the supernatants were recovered. Additional extractions were carried out with 500 µL of the mixture on the residual solids until complete depletion of pigmentation [26].

The collected extracts were concentrated to dryness under vacuum at a temperature below 30 °C using a Buchi TM R-100 (Fisher Scientific, Hampton, NH, USA). The resulting residue was reconstituted in 40 µL of ethyl acetate before instrumental analysis.

An Agilent 1200 Series Rapid Resolution Liquid Chromatography system (Agilent Technologies, Santa Clara, CA, USA), fitted with a diode array detector (DAD-UV-Vis) was employed for chromatographic separation and detection. A YMC C30 column (3 µm, 4.6 × 150 mm) (YMC Europe GmbH, Dinslaken, Germany) was used for carotenoid separation. Methanol was used as solvent A in the mobile phase composition, methyl tert-butyl ether as solvent B, and water as solvent C with a linear gradient elution as follows: 95% A + 5% B + 0% C, 0 min; 95% A + 5% B + 0% C, 5 min; 95% A + 5% B + 0% C, 5 min; 89% A + 11% B + 10% C, 10 min; 89% A + 11% B + 0% C, 10 min; 75% A + 25% B + 0% C, 16 min; 40% A + 60% B + 0% C, 20 min; 15% A + 85% B + 0% C, 22 5 min; 90% A + 5% B + 5% C, 25 min; and 90% A + 5% B + 5% C, 28 min. Identifications of carotenoids were based on matching retention times and UV-Vis spectra recorded at 350 or 450 nm.

The quantification of compounds was performed based on calibration curves constructed from individual standards solutions (1 mg/mL) of astaxanthin, lutein, zeinoxanthin, violaxanthin, zeaxanthin, α-carotene, β-carotene, β-cryptoxanthin, trans-β-apo-8-carotenal and lycopene. Results were expressed as milligrams per 100 g of dry weight (mg/100 g DW). Total carotenoid content was calculated as the sum of the quantified individual components [27].

#### 2.4.4. Phenolic Compound Identification

Phenolic compounds were extracted from 20 mg of freeze-dried powder by mixing it with 1000 µL of methanol that had been acidified with 0.1% HCl. The resulting suspension was homogenised and stirred in an ultrasonic bath for three minutes. The supernatant was recovered by centrifugation at 14,000 rpm for three minutes at 4 °C. The solid residue was then subjected to two successive extractions with 500 µL of acidified methanol [25].

Individual phenolic compounds were quantified after filtering the extract through a 0.45 µm PVDF filter. The analysis was performed on an Agilent RRLC 1200 (Agilent Technologies, USA) fitted with a DAD-UV-VIS detector operating between 220 and 500 nm, and a Zorbax Eclipse Plus C18 column (4.6 × 150 mm, 5 µm) (Agilent Technologies, USA). The mobile phase was composed of an aqueous solution containing 0.01% formic acid (solvent A) and acetonitrile (solvent B). The gradient elution was applied as follows: 100% A at 0 min, 95% A and 5% B at 5 min, 50% A and 50% B at 20 min, followed by column washing. After that, the column was rebalanced for 30 min. Then, 10 µL of each sample was injected in duplicate, and runs were monitored using Open Lab ChemStation software.

The identification of phenolic compounds was achieved through comparison of their retention times and corresponding spectral data at select wavelengths: 280 nm, 320 nm, or 370 nm, depending on the specific compound. For quantification purposes, calibration curves were generated employing 1 mg/mL of standard solution of each compound, such as gallic acid, 4-hydroxibenzoic acid, caffeic acid, *p*-hydroxybenzoic acid, 3-hydroxybenzoic acid, 2,5-dihydroxybenzoic acid, syringic acid, chlorogenic acid, ferulic acid, kaempferol, quercetin glucoside, chrysin, *p*-coumaric acid, *m*-coumaric acid, *o*-coumaric acid, luteolin, naringin, shikimic acid, vanillic acid, and quercetin. Each phenolic compound was quantified and expressed in milligrams per 100 g of dry weight (mg/100 g DW). The total phenolic content was subsequently calculated as the sum of all quantified individual phenolics.

### 2.5. Antimicrobial Activity

#### 2.5.1. Antibacterial Activity

The antibacterial activity of *D. strobilaceus* extract was assessed against ATCC strains *Escherichia coli* ATCC 8739, *Pseudomonas aeruginosa* ATCC 9027, *Staphylococcus aureus* ATCC 6538P, and *Streptococcus mutans* ATCC 25175, sourced from the American Type Culture Collection (ATCC, Manassas, VA, USA).

To obtain an extract rich in phenolic compounds, 2.5 g of lyophilised powder from each sample was suspended in 25 mL of 50% ethanol and homogenised, followed by sonication in an F560 ultrasonic bath (Scietific, Waltham, MA, USA) for 6 min to enhance extraction efficiency. The mixture was then centrifuged at 7500 rpm for 5 min at 4 °C using a microcentrifuge (Eppendorf, Bochum, Germany), and the supernatant was collected. The extraction procedure was repeated twice more, with 25 mL of the ethanol solution added each time to ensure complete recovery of the target compounds. The combined supernatants were filtered through 0.45 µm × 25 mm PVDF syringe filters. Ethanol was removed by rotary evaporation at temperatures not exceeding 40 °C. The remaining material was frozen. Extracts were subsequently dried using a Christ Alpha 1–4 LDplus freeze dryer (GmbH, Bochum, Germany), and then lyophilized. Afterwards, the dried extract was stored under frozen conditions until further analysis. Finally, the resulting dry residue 300 mg was reconstituted in 2 mL of sterile distilled water [28]. The initial concentration was selected based on the solubility of the extract and to encompass a wide range of potentially active doses, MIC values for crude plant extracts could be within the milligram per millilitre scale [29].

Determination of the minimal inhibitory concentration (MIC) was carried out using the microdilution technique in accordance with the Clinical and Laboratory Standards Institute (CLSI) recommendations [30,31]. Bacterial strains were pre-cultivated in brain heart infusion (BHI) broth and incubated overnight at 37 °C with continuous agitation on a rotary shaker. Subsequently, 100 µL of each extract solution was mixed with 100 µL of Brain Heart Infusion (BHI) medium, and serial dilutions were carried out directly in the microplate. Then, 20 µL of the bacterial suspension was added to each well, adjusted to a final concentration of 5 × 10^5^ CFU/mL, resulting in a total reaction volume of 220 µL in the microplate. Streptomycin was used as a positive control, whereas the negative control corresponded to the microorganism suspension with water. The microplates were incubated at 37 °C for 20 h. To assess microbial growth, 20 µL of 2,3,5-triphenyltetrazolium chloride (TTC) was added, followed by incubation at 37 °C for an additional two hours. Metabolically active bacteria reduce TTC to a red-coloured formazan, which enables the straightforward visual detection of bacterial growth inhibition. The minimal inhibitory concentration (MIC) was determined by analysing the growth inhibition profile over time in samples exposed to varying concentrations of extracts. All assays were conducted at least in triplicate.

##### Antibacterial Activity for Multidrug-Resistant Bacteria

Antibacterial activity of the *D. strobilaceus* extract was determined by the microdilution assay against seven multidrug-resistant (MDR) bacterial strains such as *Klebsiella pneumoniae*, *Escherichia coli*, *Salmonella enterica* serovar Kentucky, *Enterococcus faecalis*, *Staphylococcus epidermidis*, *Enterococcus faecium*, and *Pseudomonas aeruginosa* using the microdilution method. These clinical MDR isolates were obtained from the National Institute of Public Health of Ecuador (INSPI). These strains had been previously identified and characterised by INSPI. The clinical strains correspond to isolates that have also been part of the External Quality Evaluation Program of INSPI.

Bacterial inocula were prepared in brain–heart infusion (BHI) broth to achieve a final cell density of 5 × 10^5^ CFU/mL. The microbial density was adjusted spectrophotometrically. The tested compound (Section 2.5.1) was dissolved in water to prepare a stock solution at a concentration of 257 mg/mL. Additionally, nourseothricin (100 µg/mL) (Goldbio, St. Louis, MO, USA) was employed as a growth inhibition control. Blank samples consisted of BHI medium alone and BHI containing the extract at stock concentration served as blanks.

The antibacterial sensitivity of the extracts was assessed following the microdilution procedure described by the Clinical and Laboratory Standards Institute (CLSI), with slight modifications. Briefly, 5 µL of the extract stock solution was combined with 195 µL of a bacterial suspension (5 × 10^5^ CFU/mL), yielding a final volume of 200 µL. Microplates were incubated at 37 °C for 20 h under constant agitation at 300 cpm (double orbital setting). The MIC values were established by comparing the optical density at 600 nm at the beginning and after 24 h of incubation for treated samples and both positive and negative controls. Each experiment was conducted in triplicate.

#### 2.5.2. Antifungal Activity

Determination of the minimum inhibitory concentration (MIC) was performed using the microdilution technique according to the Clinical and Laboratory Standards Institute (CLSI) guidelines [30,31,32]. The yeast strains used in this study were *Candida albicans* ATCC 1031 and *Candida tropicalis* ATCC 13803 obtained from the American Type Culture Collection.

For fungal inoculum, cultures were obtained by pre—cultivating yeast peptone dextrose broth (YPDB). The stock solution of the tested extract was obtained by dissolving 300 mg of the lyophilised material (Section 2.5.1) in 2 mL of sterile distilled water. Subsequently, 100 µL of each extract solution was mixed with 100 µL of yeast peptone dextrose broth (YPDB), and serial dilutions were carried out directly in the microplate. Then, 20 µL of the bacterial suspension was added to each well, adjusted to a final concentration of 1 × 10^6^ CFU/mL, resulting in a total reaction volume of 220 µL in the microplate Fluconazole was used as a positive control. In contrast, the negative control consisted of suspending the microorganism in water. The microplates were incubated at 37 °C for 72 h. The minimal inhibitory concentration (MIC) was determined by analysing the growth inhibition profile over time in samples exposed to varying concentrations of extracts. All assays were conducted at least in triplicate.

### 2.6. Antioxidant Activity

Antioxidant activity was assessed using two complementary methods, such as DPPH and ABTS. A 20 mg portion of the freeze-dried sample was combined with 2 mL of methanol and homogenised using a vortex mixer. Ultrasonication was applied for 3 min to facilitate compound extraction. After phase separation by centrifugation at 4 °C and 131,171× *g* for 3 min, the clear supernatant was collected, passed through a 0.45 µm PVDF filter and subsequently used for analysis [21,33].

To prepare the calibration curve, 10 mM a stock solution of Trolox between 0.4 and 4 mM was prepared in methanol. To prepare the DPPH^•^ radical solution, 10 mg of DPPH was dissolved in 50 mL of methanol. In the assay, 20 µL of sample or standard was mixed with 280 µL of freshly prepared DPPH solution in a 96-well VMT microplate (Corning, Glendale, AZ, USA). The microplate was incubated for 30 min in the dark on Shaker 4310 orbital platform (Fisher Scientific, Waltham, MA, USA), after which the absorbance was recorded at 515 nm using a BioTek H1 spectrophotometer (Agilent Scientific Instrument, Santa Clara, CA, USA). The antioxidant activity was expressed in millimoles of Trolox equivalents per 100 g of dry weight (mmol TE/100 g DW).

To generate the ABTS^•+^ radical, equal volumes of 7 mM ABTS and 0.45 nM potassium persulfate solutions were combined, followed by incubation in the dark for 16 h. Before use, the ABTS^•+^ radical was diluted tenfold with absolute ethanol to achieve an absorbance of approximately 0.7 at 734 nm. A Trolox calibration curve was constructed using a 10 mM stock solution diluted between 0.2 and 0.7 mM in ethanol. In each well of a 96-well VWT microplate, 20 μL of sample and 280 μL of ABTS^•+^ working solution were added. Absorbance readings were taken at 734 nm using a Thermo Scientific Multiskan GO spectrophotometer (Agilent Scientific Instruments, Santa Clara, CA, USA). Antioxidant capacity was expressed as millimoles of Trolox equivalents per 100 g of dry weight (mmol TE/100 g DW).

### 2.7. Anticancer Activity

HeLa (human cervical carcinoma, ATCC No. CCL-2, RRID: CVCL_0030), HCT116 (human colorectal carcinoma (human breast adenocarcinoma, ATCC No. CCL-247, RRID: CVCL_0291), HepG2 (human hepatoma, ATCC No. HB-8065, RRID: CVCL_0027), and NIH3T3 (mouse NIH/Swiss embryo fibroblasts, ATCC No. CRL-1658, RRID: CVCL_0594) cell lines were obtained from the American Type Culture Collection (ATCC, Manassas, VA, USA). THJ29T (human thyroid carcinoma, Cat. No. T8254, RRID: CVCL_W922) was obtained from Applied Biological Materials Inc. (ABM, Richmond, BC, Canada). This selection was made to encompass diverse tissues of origin and molecular backgrounds, allowing for a broader assessment of the antiproliferative activity of the extracts. The culture media selected for this study are standard and widely used for these cell lines, as they fully meet their growth requirements. All cell lines were cultured in Dulbecco’s Modified Eagle’s Medium/Nutrient Mixture F-12 Ham (DMEM/F12) ( Corning, VA, USA) supplemented with 10% foetal bovine serum (FBS) (Eurobio, Les Ulis, France) and 1% penicillin/streptomycin (Thermo Fisher Scientific, Gibco, Miami, FL, USA) and maintained at 37 °C in a humidified atmosphere with 5% CO_2_.

To investigate the effect of *D. strobilaceus* extract on cellular proliferation, cells were seeded in 96-well plates at a density of 1 × 10^4^ cells per well. After a 24 h attachment period, cells were treated for 72 h with 100 µL of the extract prepared in distilled water using serial two-fold dilutions ranging from 0.01 mg/mL to 2.5 mg/mL, a concentration range determined based on the maximum solubility of the extract and the highest non-toxic amount of solvent that could be safely added to the cells, as described in Section 2.5.1. Following the incubation period, cell proliferaton was assessed using the MTT assay following standard protocols. Briefly, 10 μL of MTT (M5655, Sigma, Ronkonkoma, NY, USA) solution (5 mg/mL in PBS) was added to each well, and the plates were incubated for 1–2 h at 37 °C in a humidified atmosphere. Subsequently, the supernatant was carefully aspirated, and 50 μL of DMSO was added to each well to dissolve the resulting formazan crystals. The plates were gently agitated for 2 min to ensure complete solubilization before measuring absorbance at 570 nm using the Cytation 5 multi-mode detection system (BioTek, Winooski, VT, USA). Each concentration was tested in quadruplicate across a minimum of four independent experiments, with results expressed as mean ± standard deviation. Media alone was included as a negative control, representing 100% cell proliferation, and the percentage of inhibition was calculated relative to this control. To determine the concentration required to inhibit 50% of cell proliferation (IC_50_), dose–response curves were generated using GraphPad Prism 10.2 (GraphPad Software, Inc., San Diego, CA, USA). The therapeutic index (TI) was calculated by dividing the IC_50_ value obtained for non-tumour cells (NIH3T3) by that of each tumour cell line. Cisplatin (CDDP) was used as a positive control at 0.1–10 μg/mL, and IC_50_ values were calculated from the dose–response curves.

### 2.8. Anti-Inflammatory Activity

RAW 264.7 murine macrophages (ATCC TIB-71, RRID: CVCL_0493) were cultured in DMEM (4.5 g/L glucose, *L*-glutamine; Corning, USA) supplemented with 10% foetal bovine serum (Eurobio, Les Ulis, France) and 1% penicillin-streptomycin (Thermo Fisher Scientific, USA). Routine maintenance was performed as described by Wallert et al. [34], using 30% conditioned medium (DMEM obtained from a previous passage of RAW264.7 macrophage culture) and 70% fresh medium. Cultures were incubated at 37 °C in 5% CO_2_.

For the anti-inflammatory assay, two concentrations of the extract (0.025 and 0.050 mg/mL) were selected based on the sensitivity of the cells (RAW264.7) and the nature of the assay. The IC_50_ in these cells was calculated using the MTT assay, yielding a value of 0.060 mg/mL. These lower concentrations were chosen to avoid cytotoxic effects while still allowing detection of potential anti-inflammatory activity. 3 × 10^5^ cells/well were seeded into 24-well plates and allowed to adhere for 18–24 h. The medium was then replaced with serum-free DMEM containing: control (DMEM), positive control (dexamethasone, 2 μg/mL; Sigma-Aldrich, USA), or *D. strobilaceus* extract (0.025 or 0.05 mg/mL). After 4 h, lipopolysaccharide (LPS, 1 μg/mL; InvivoGen, Toulouse, France) was added to stimulate nitric oxide (NO) production, and incubation continued for 20 h. Stock solutions were prepared in DMEM. Supernatants (50 μL) were collected and mixed with 50 μL of Griess reagent (Sigma-Aldrich, 50 mg/mL) in 96-well plates. A NO standard curve (0.78–100 μM; Promega, Madison, WI, USA) was included. Following a 10 min incubation at room temperature under dark conditions, absorbance readings were taken at 540 nm (Cytation 5, BioTek). NO levels were calculated from the calibration curve after background subtraction. Assays were performed in triplicate. Cell viability was assessed in parallel by fixing cells with 4% paraformaldehyde for 20 min, staining with 0.5% crystal violet for 30 min, washing, and air-drying. Plates were scanned, and absorbance at 570 nm was quantified (Cytation 5, BioTek). Untreated cells served as a viability reference (100%).

### 2.9. Statistical Analysis

Statistical analyses were conducted using RStudio version 4.4.1, STATGRAPHICS Centurion XVII, and SigmaPlot 14.0. The results are presented as mean ± standard deviation. A one-way analysis of variance (ANOVA) was applied, followed by Tukey’s post hoc test for mean comparisons, with statistical significance set at *p* < 0.05. At a 95% confidence level, Pearson’s correlation analysis was performed to determine the relationships between the studied parameters.

## 3. Results

### 3.1. Physico-Chemical Analysis

Traditionally, plant species, including their leaves, stems, roots, and flowers, have been used for medicinal purposes. Thus, Table 1 shows the average physicochemical properties of ‘Caña agria’, including pH, soluble solids, titratable acidity, moisture content and ash content in the leaves, stems, and seeds. It also shows the mineral content, including calcium, iron, potassium, magnesium, and sodium.

### 3.2. Bioactive Compounds

Plants are widely recognised as containing various bioactive compounds distributed throughout their different structures. Table 2 presents the results of the phytochemical screening of different groups of organic molecules found in this study. In contrast, Table 3 shows the average values for the vitamin C concentration, organic acid profile, carotenoids, chlorophylls and their derivatives and phenolic compounds of ‘Caña agria’ leaves, stems, and seeds.

### 3.3. Antimicrobial Activity

The presence of bioactive compounds in particular plant species confers antimicrobial activity against various pathogens. Table 4 presents the minimum inhibitory concentration (MIC) values obtained against bacteria, including *Escherichia coli*, *Pseudomonas aeruginosa*, *Staphylococcus aureus*, and *Streptococcus mutans*; *Candida albicans* and *Candida tropicalis* and the multidrug and non-multidrug resistant bacteria. Although these values are higher than those reported for pure antimicrobial agents, such differences are expected for crude extracts, in which the active fraction constitutes only a small proportion of the total material. The relative efficacy of the extract, therefore, refers to its inhibitory potential compared with standard antibiotics, rather than to an equivalent concentration response. The extract at the maximum concentration tested (6.42 mg/mL) did not show any antibacterial effect for multidrug-resistant bacteria.

### 3.4. Antioxidant Activity

Plant species are characterised by the presence of secondary metabolites, which give them significant antioxidant properties. The antioxidant activity results obtained from the DPPH and ABTS assays are summarised in Table 5.

### 3.5. Anticancer Activity

The antiproliferative activity of Caña agria leaf extract was evaluated by MTT assay in a panel of tumour cell lines (HeLa, HCT116, THJ29T, and HepG2) alongside non-tumour NIH3T3 fibroblasts (Table 6).

The extract exhibited cytotoxic effects in all tumour cell lines tested, with IC_50_ values falling below 0.2 mg/mL, indicating moderate potency for a crude extract [35]. HepG2 and THJ29T cells were the most sensitive, while HCT116 cells showed slightly reduced susceptibility. Therapeutic index (TI) values ranged from 0.8 to 1.8, suggesting modest selectivity. The most favourable TI was observed in hepatoma cells (HepG2), followed by thyroid (THJ29T) and cervical carcinoma cells (HeLa). In contrast, the extract’s activity against colorectal carcinoma cells was less selective, with the TI falling below 1.0. For comparison, the positive control cisplatin (CDDP) exhibited consistently lower IC_50_ values than Caña agria leaf extract, confirming its expected antiproliferative activity, while maintaining low TI values across the cell lines, indicative of limited selectivity.

### 3.6. Anti-Inflammatory Activity

**Figure 2:**
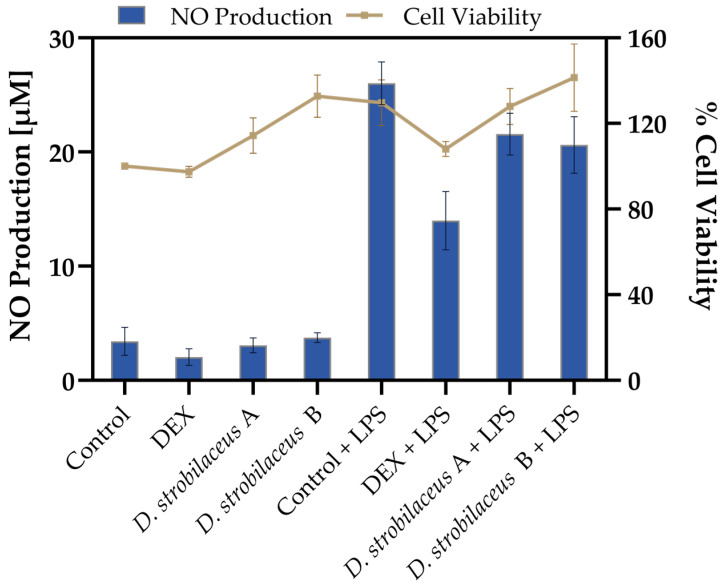
Effects of *D. strobilaceus* leaf extract (A = 0.025 or B = 0.05 mg/mL) on RAW264.7 macrophages. Nitric oxide (NO) production (left *y*-axis, bars) and cell viability (right *y*-axis, line) under the same conditions. Data are expressed as mean ± SD (n = 3).

To evaluate the effects of *D. strobilaceus* leaf extract, RAW 264.7 macrophages were exposed to the extract in the presence or absence of LPS stimulation. As shown in Figure 2, basal NO production in untreated cells was 3.4 ± 1.2 µM, and in all non-stimulated conditions, NO levels remained below 4 µM. Upon LPS stimulation, NO levels increased markedly to 26.0 ± 1.9 µM. DEX significantly reduced this induction to 14.0 ± 2.6 µM. *D. strobilaceus* leaf extract also attenuated LPS-induced NO production, yielding 21.6 ± 1.8 µM (0.025 mg/mL) and 20.6 ± 2.5 µM (0.05 mg/mL), although this reduction was less pronounced than that achieved with DEX.

Cell viability results (Figure 2) showed values ranging from 97.4 ± 2.6% to 141.4 ± 15.7% across all tested conditions, indicating that treatments with *D. strobilaceus*, DEX, and LPS maintained viable cell populations. Additionally, no morphological alterations were observed under any condition shown.

## 4. Discussion

### 4.1. Physico-Chemical Analysis

*Dimerocostus strobilaceus*, commonly known as ‘caña agria’, is a plant species that has been traditionally used in Ecuadorian folk medicine to treat digestive disorders and prostate cancer. This study characterised its physicochemical properties, revealing significant variability between different parts of the plant. This suggests that leaves, stems, and seeds have different functions and metabolic processes.

In terms of pH, the observed range was between 4.2 in the seeds and 6.1 in the stems, reflecting the physiological differences between the plant organs. The seeds exhibited a more acidic pH, as would be expected given that they are storage structures with low metabolic activity. Their primary function is to protect the embryo until germination, thereby maintaining a more controlled and chemically stable internal environment [36]. Conversely, the stems had the highest pH in the study. As they serve as a transport route for water and nutrients, stems must maintain relative neutrality to ensure the vascular system functions properly. This pH stability also contributes to structural integrity and the efficient exchange of substances between the roots and leaves. In contrast, the leaves exhibited higher pH values, which are associated with their high physiological and metabolic activity. Not only do leaves perform photosynthesis, but they also interact directly with the environment through their surface, which can modify pH in response to external conditions. This phenomenon, known as phylloplane pH, varies depending on species, water availability, nutrients and the level of environmental stress. Furthermore, factors such as salinity or drought can disrupt the internal balance of leaf cells, reducing pH and affecting vital processes, including photosynthesis and water regulation [37,38].

Regarding soluble solids content, low values were recorded, ranging from 0.1 °Brix in leaves and seeds to 0.5 °Brix in stems. These results reflect the low levels of free sugars or dissolved solutes characteristic of non-fruit species or vegetative structures, which do not primarily store energy reserves [39].

Titratable acidity was consistent at 0.2% across all analysed plant parts. This stability can be interpreted as an indicator of the balanced presence of organic acids, which play essential roles in intermediate metabolism, cellular pH regulation, and the response to oxidative stress. The concentration of compounds such as citric, malic, and oxalic acids directly influences titratable acidity in plant tissues, and these acids also participate in key pathways, including the tricarboxylic acid (TCA) cycle, C4 photosynthesis, and heavy metal chelation [40].

The moisture content ranged from 76.8% in the stems to 80.4% in the leaves. This indicates that all of the analysed structures have a high-water content, as would be expected of perennial herbaceous species adapted to tropical environments. As highly photosynthetic tissues, leaves must maintain high turgor pressure to facilitate stomatal opening and CO_2_ absorption. Meanwhile, although the stems are also watery, they have lower relative humidity, which could be related to their structural and support function [41].

Ash content varied from 1.6% in stems to 2.7% in leaves, indicating a higher mineral concentration in the leaves. This finding is consistent with the tissue’s high metabolic rate, which requires constant input and recycling of macro- and micronutrients for photosynthesis, oxidative defence, and production of secondary metabolites [42].

A clear differential distribution of macro- and micronutrients was observed in the different parts of *D. strobilaceus*. The leaves had the highest concentrations of all analysed minerals, followed by the stems and then the seeds. This reflects their highly active physiological function and direct exposure to the environment.

Potassium was the predominant mineral in all tissues, ranging from 981.3 mg/100 g DW in the seeds to 4074.8 mg/100 g DW in the leaves. This is consistent with potassium’s key roles in osmotic regulation, stomatal opening, enzyme activation, and the transport of photoassimilates. Given the intense photosynthetic activity and the need to maintain cellular water balance in this tissue, its high concentration in leaves is to be expected [42]. The World Health Organisation (WHO) recommends a daily potassium intake of over 3500 mg for adults to reduce the risk of hypertension and cardiovascular disease [43]. Thus, the potassium content recorded in *D. strobilaceus* leaves suggests their high potential for use as a natural source of potassium in functional formulations.

Calcium was found to be the second most abundant element, with concentrations ranging from 341.4 mg/100 g DW in seeds to 3559.5 mg/100 g DW in leaves. It is essential for cell wall structure, controlling cell growth and intracellular signalling. Its high accumulation in leaves suggests a significant structural function in photosynthetic tissues that are subject to environmental fluctuations [43]. In terms of nutrition, adequate calcium intake (1000–1200 mg per day for adults) has been linked to the prevention of bone diseases such as osteoporosis and the reduction in kidney stone formation by regulating oxalate absorption in the gastrointestinal tract [44].

There was also an increasing trend in iron levels from seeds (42.5 mg/100 g DW) to leaves (291.5 mg/100 g DW), which supports the idea that metabolically active tissues accumulate higher levels of this mineral. Magnesium values ranged from 120.0 mg/100 g DW in seeds to 390.3 mg/100 g DW in leaves, while sodium concentrations were lower and less variable between organs, ranging from 11.7 mg/100 g DW in stems to 17.8 mg/100 g DW in seeds. Although sodium is not considered essential for most plants, its presence in low concentrations can contribute to maintaining ionic balance and osmotic stability. From a human health perspective, a low sodium concentration in plant products is desirable as high intake of this mineral is associated with an increased risk of hypertension [45].

Overall, the mineral profile of *Dimerocostus strobilaceus* exhibits a pattern commonly observed in tropical species, where the leaves serve as reservoirs for vital nutrients. Agronomic and environmental factors, such as soil type, water availability, pH, and interactions with other nutrients, may influence this accumulation. Previous studies, such as those conducted on *Rhus verniciflua*, have demonstrated that mineral distribution can remain constant across tissues. However, there are also species in which seeds concentrate potassium or magnesium at significant levels, broadening their applications in the food industry [42,46].

Ultimately, it is essential to recognise that the mineral composition of plants can vary substantially depending on their ecological environment. For instance, a copper deficiency in poplars can significantly impact the absorption and distribution of other vital nutrients, highlighting the intricate relationships between soil, environment, and plant physiology [46].

### 4.2. Bioactive Compounds

Secondary metabolites in plants are the products of specialised metabolism. They do not directly participate in growth or reproduction, but they perform key functions in adaptation to the environment and defence against pathogens. In many cases, they also offer benefits to human health [47,48]. In the present study, *Dimerocostus strobilaceus* exhibited a diverse biochemical profile, with significant variations in metabolite distribution between leaves, stems, and seeds.

One of the most potent antioxidants in biological systems, vitamin C (ascorbic acid), showed wide variability between organs, ranging from 0.4 mg/100 g DW in stems to 16.7 mg/100 g DW in leaves. The higher concentration in leaves can be attributed to their intense photosynthetic activity and direct exposure to oxidative stress, justifying the need to accumulate protective compounds. This pattern has also been reported in other species, where leaves concentrate more vitamin C than seeds and flowers [49]. From a nutritional perspective, although levels in *D. strobilaceus* are moderate, they could contribute to the EFSA’s recommended daily intake of 110 mg for men and 95 mg for women [50], particularly when considering potential consumption as an extract or functional ingredient.

Organic acids, such as malic, citric, and tartaric acids, are key compounds in intermediate metabolism and cellular pH regulation. The total content of organic acids ranged from 744.9 mg/100 g DW in seeds to 799.8 mg/100 g DW in leaves, with malic acid predominating, followed by citric acid. The content of citric acid varied from 117.7 mg/100 g DW in stems to 196.7 mg/100 g DW in leaves, while the content of malic acid ranged from 551.9 mg/100 g DW in leaves to 575.9 mg/100 g DW in seeds, and the content of tartaric acid ranged from 42.7 mg/100 g DW in seeds to 76.9 mg/100 g DW in stems. The relatively uniform distribution of these compounds suggests a shared metabolic function among tissues, albeit with slight variations that reflect differences in respiratory activity and cellular maturity. These compounds participate in fundamental biochemical pathways, such as the tricarboxylic acid cycle, and contribute to the sour taste and preservation of plant products [40].

Six main carotenoid compounds were identified, such as lutein, zeinoxanthin, violaxanthin, zeaxanthin, α-carotene, and β-carotene. Their total concentration ranged from 0.5 mg/100 g DW in seeds to 356.4 mg/100 g DW in leaves. The higher accumulation in leaves is consistent with their role in capturing light and protecting against photo-oxidative damage. Lutein ranged from 0.4 mg/100 g DW in seeds to 65.3 mg/100 g DW in leaves; zeinoxanthin was only present in leaves (2.3 mg/100 g DW); violaxanthin ranged from 0.1 mg/100 g DW in seeds to 15.mg/100 g DW (leaves); zeaxanthin was present in the leaves (2.1 mg/100 g DW) and the stems (0.1 mg/100 g DW); α-carotene was only present in the leaves (4.9 mg/100 g DW); and β-carotene was present in the leaves (266.6 mg/100 g DW) and the stems (1.1 mg/100 g DW). Notably, β-carotene was present at a concentration of 266.6 mg/100 g DW in leaves, which highlights the functional value of this tissue as a source of provitamin A.

A higher concentration of chlorophylls and their derivatives was found in the leaves (123.3 mg/100 g DW) than in the stems and seeds. The main pigment was chlorophyll b (87.9 mg/100 g DW in leaves), followed by pheophytin (35.4 mg/100 g DW). This result is to be expected, given that leaves are the main site of photosynthesis. The partial conversion of chlorophyll to pheophytin suggests an active degradation or recycling process, which is common in tissues that experience fluctuations in light or undergo cellular ageing. While there is no direct conversion between chlorophyll and carotenoids, the two groups of compounds are metabolically linked and work together to protect against reactive oxygen species during photosynthesis [51,52].

Phenolic compounds exhibited the most significant variability between organs of all the metabolites. The following were identified: gallic acid, 4-hydroxybenzoic acid, caffeic acid, syringic acid, chlorogenic acid, ferulic acid, kaempferol, quercetin glucoside and quercetin. The total phenol content ranged from 195.4 mg/100 g DW in stems to 3658.5 mg/100 g DW in seeds. The particularly high concentration in seeds is noteworthy, as these compounds contribute to defending the embryo against oxidative stress and microbial attacks [36]. Gallic acid varied from 8.4 mg/100 g DW in seeds to 39.8 mg/100 g DW in leaves; 4-hydroxybenzoic acid ranged from 34.9 mg/100 g DW in leaves to 44.6 mg/100 g DW in seeds; and caffeic acid ranged from 27.1 mg/100 g DW in leaves to 1325.4 mg/100 g DW in seeds. Syringic acid ranged from 19.1 mg/100 g DW in seeds to 154 mg/100 g DW. Chlorogenic acid was found only in the leaves (35.7 mg/100 g DW) and seeds (252.7 mg/100 g DW), while ferulic acid was only found in the leaves (75.9 mg/100 g DW) and seeds (2008.3 mg/100 g DW). Kaempferol (101.8 mg/100 g DW), quercetin glucoside (54.5 mg/100 g DW) and quercetin (34.7 mg/100 g DW) were only found in the leaves.

The phenolic composition of the stems was significantly lower, which may be related to their lower metabolic activity and structural function. However, these compounds (caffeic and ferulic acid) could contribute to the observed antioxidant and antimicrobial properties of this tissue. Similar distributions have been reported in previous studies, such as in *Rhus verniciflua*, with higher concentrations of polyphenols and flavonoids found in shoots and leaves compared to stems and seeds [46].

### 4.3. Antimicrobial Activity

Secondary metabolites such as phenolic compounds, organic acids, flavonoids, and carotenoids, in plants has been linked to their remarkable antimicrobial capacity. These metabolites act as natural defence mechanisms against bacteria and fungi [27,53]. In the case of *Dimerocostus strobilaceus*, differential antimicrobial activity was observed in different plant structures, indicating the specific distribution of inhibitory metabolites.

The minimum inhibitory concentration (MIC) against Gram-negative and Gram-positive bacteria varies according to the microorganism and plant part evaluated. For *Escherichia coli*, an MIC of 10.5 mg/mL was observed in stems and seeds, whereas inhibition was achieved at a higher concentration (20.8 mg/mL) in leaves. This suggests that the antimicrobial compounds present in stems and seeds may be more concentrated or effective against this enteric pathogen. According to clinical criteria, an antimicrobial is considered effective against *E. coli* when its MIC is ≤16 mg/L, as is the case with drugs such as levofloxacin [54].

For the notoriously resistant bacterium *Pseudomonas aeruginosa*, an MIC of 41.8 mg/mL was recorded in both stems and seeds. While this concentration is higher than that observed against *E. coli*, it suggests that *D. strobilaceus* extracts contain compounds that can inhibit multidrug-resistant bacteria. This is significant given the lack of effective treatments for this species. A uniform MIC of 41.8 mg/mL was observed in leaves, stems, and seeds of *S. aureus*, suggesting that the active compounds against this bacterium are distributed throughout the plant, albeit in moderate concentrations.

Interestingly, *Streptococcus mutans*, a bacterium associated with oral diseases such as dental caries, was sensitive only to the leaf extract, with an MIC of 5.2 mg/mL. This value indicates considerable antimicrobial activity, which may be attributed to the high concentration of specific phenolic compounds in the leaves, such as gallic acid, quercetin, and kaempferol, known to inhibit oral bacteria.

As for the fungi, the antifungal activity was more evident in the leaves. *Candida albicans* and *C. tropicalis* both showed an MIC of 10.4 mg/mL in leaves, whereas higher concentrations (41.8 mg/mL) were needed to inhibit growth in stems and seeds. These results suggest that compounds with antifungal properties are concentrated preferentially in leaf tissue, probably as an adaptive response to environments with high microbial loads. The action of phenolic compounds and specific organic acids may be involved in this response.

Future studies should include characterisation or fractionation of the active components to establish a more direct comparison with recognised antimicrobial standards.

The lack of measurable antibacterial activity of *D. strobilaceus* extracts against MDR isolates could be explained by the intrinsic pharmacodynamic and permeability barriers to these pathogens. In Gram-negative strains, the lipopolysaccharide outer membrane and overexpressed efflux pumps could restrict the intracellular accumulation of phytochemicals to subinhibitory levels [55,56]. Simultaneously, robust biofilm formation and stress-response networks could attenuate redox or membrane perturbations typical of polyphenols [57], explaining the lack of detectable inhibition in conventional assays.

Additionally, several studies have demonstrated that the antimicrobial activity of organic acids is affected by the pH of the medium. For example, compounds such as ascorbic acid and malic acid are more effective in acidic environments as their undissociated form can cross the bacterial cell membrane, releasing protons into the cytoplasm and altering the internal balance [58]. In *D. strobilaceus*, where an acidic pH was observed, especially in seeds and leaves, this effect could enhance the action of the present organic acids. Furthermore, the synergistic combination of these acids has been documented to reduce the minimum inhibitory concentration (MIC) significantly required to exert an antimicrobial effect, opening up possibilities for future studies on combinations of extracts or active fractions [59].

### 4.4. Antioxidant Activity

The antioxidant capacity of *D. strobilaceus* is directly related to the presence of secondary metabolites, such as phenolic compounds and carotenoids. These compounds play a fundamental role in neutralising reactive oxygen species (ROS) and preventing oxidative damage to plant and animal cells. In the present study, antioxidant activity was evaluated using the widely employed DPPH and ABTS methods, which provide complementary information due to their differing sensitivities and mechanisms of action [33].

Using the DPPH method, values were obtained ranging from 4.3 to 5.0 mmol TE/100 g DW for stems and from 3.4 to 6.0 mmol TE/100 g DW for seeds. The ABTS assay recorded a wider range of values: from 3.4 to 6.0 mmol TE/100 g DW for stems and from 4.3 to 5.0 mmol TE/100 g DW for seeds. The seeds exhibited the highest activity in both assays, consistent with their high concentration of total phenolic compounds, particularly ferulic acid and caffeic acid, whose antioxidant efficacy is well documented. These compounds act as hydrogen or electron donors, stabilising free radicals and preventing the spread of oxidative reactions in biological systems. Although both the DPPH and ABTS assays are based on the ability of antioxidants to reduce free radicals, they respond differently depending on the nature of the compounds. The DPPH radical is lipophilic and responds better to hydrophobic compounds, such as certain carotenoids. In contrast, ABTS can interact with both lipophilic and hydrophilic compounds, thus offering a more comprehensive assessment of the antioxidant potential of complex extracts [60,61].

In this context, the phenolic compounds identified in *D. strobilaceus*, including hydroxybenzoic acids (such as gallic and protocatechuic acids), hydroxycinnamic acids (such as ferulic, caffeic and chlorogenic acids), flavonoids (such as quercetin and kaempferol), and other derivatives, represent a chemically diverse group with high reducing potential. The antioxidant efficacy of these compounds depends not only on their presence but also on the synergy between them. Indeed, various studies have demonstrated that interactions between phenolic compounds can either enhance or inhibit antioxidant capacity, depending on their structural conformation and relative concentration in the extract [62,63].

Furthermore, carotenoids contribute significantly to the observed antioxidant activity, primarily through their ability to eliminate singlet oxygen and peroxyl radicals. High concentrations of β-carotene, lutein, and violaxanthin were found in the leaves *of D. strobilaceus*, which are known for their protective action against reactive oxygen species generated under light stress conditions. Among these, β-carotene is a potent scavenger of lipophilic radicals, while lutein effectively protects lipids from peroxidation in cell membranes [64]. However, it should be noted that the antioxidant efficacy of carotenoids may be influenced by structural factors, such as the length of the conjugated chain and the presence of oxygenated functional groups, as well as by their interaction with other antioxidants, including vitamin C and polyphenols [65].

### 4.5. Anticancer Activity

Leaves of *D. strobilaceus* were selected for antiproliferative testing based on their rich content of bioactive compounds—including flavonoids (kaempferol, quercetin derivatives), carotenoids, phenolic acids, and vitamin C—which were largely absent or present at much lower levels in the seeds. This study is the first to report the antiproliferative effects of *D. strobilaceus* leaf extract in tumour cells. While this species has been used in traditional medicine for inflammatory and gastrointestinal conditions, its anticancer potential has not been previously explored. In contrast, related species from the Costaceae family, particularly those from the Costus genus, have shown cytotoxic activity in vitro [66,67,68]. Extracts from Costus speciosus and Costus afer have demonstrated antiproliferative effects in various cancer cell lines, with IC_50_ values typically ranging from 0.2 to 0.6 mg/mL. These effects have been attributed to secondary metabolites such as steroidal saponins and flavonoids. The antiproliferative activity observed in *D. strobilaceus* appears comparable in potency, despite the use of a crude extract.

Although antioxidant activity was detected, it was relatively low, indicating that redox modulation is unlikely to be the main mechanism underlying the extract’s antiproliferative effects. Instead, the phytochemical profile revealed high levels of phenolic acids and flavonoids, such as kaempferol, ferulic acid, and quercetin derivatives, which are well-documented for their ability to interfere with cancer cell proliferation and survival pathways [69,70]. The extract also contained high levels of organic acids, particularly malic and citric acid. However, their contribution to the antiproliferative effect is likely limited and secondary to that of phenolic compounds.

Despite the promising potency observed, the TI values indicate only moderate selectivity, similar to what is observed for cisplatin, suggesting that the extract exerts cytotoxic effects on both tumour and non-tumour cells at comparable concentrations. Nonetheless, the variable sensitivity among tumour cell lines likely reflects differences in transporter expression, metabolic enzyme activity, and signalling networks that influence the uptake, bioavailability, and intracellular action of the phenolic and flavonoid constituents. The higher selectivity in hepatocellular carcinoma cells suggests that the extract may preferentially target pathways more active in hepatic tumour cells compared to the broad DNA-damaging activity of cisplatin. This enhanced sensitivity may be related to the elevated metabolic rate and dependence of these cells on redox-regulated proliferative signalling, which can be disrupted by phenolic compounds even when overall antioxidant capacity is low. This suggests that the extract’s antiproliferative effects are not primarily due to direct ROS scavenging but to interference with redox-sensitive signalling networks, consistent with the known mechanisms of kaempferol and quercetin derivatives [71,72]. This highlights the importance of future studies aimed at isolating active constituents or employing targeted delivery approaches to improve tumour specificity. Altogether, these results position *D. strobilaceus* as a previously uncharacterised but promising source of bioactive metabolites with antiproliferative potential, warranting further investigation through compound fractionation and mechanistic evaluation.

### 4.6. Anti-Inflammatory Activity

The leaf extract of *D. strobilaceus* moderately suppressed nitric oxide (NO) production in LPS-stimulated RAW 264.7 macrophages, reducing levels by 16–21% at 0.025–0.05 mg/mL, compared with 46% inhibition by dexamethasone (basal NO production subtracted). This effect may be attributed to the distinct composition and abundance of anti-inflammatory secondary metabolites in the leaves compared with other organs, particularly vitamin C, carotenoids, flavonoids, and phenolic acids. Among these, quercetin and kaempferol are especially relevant, as they are known to downregulate COX-2 and iNOS expression [73,74]. Additionally, phenolic acids, including gallic, ferulic, caffeic, and chlorogenic acids, could contribute to the anti-inflammatory activity [75]. In vivo studies have shown that gallic and taurine acids increase IL-10 levels while reducing NF-κB and IL-1β expression, demonstrating synergistic anti-inflammatory effects [76]. Carotenoids abundant in leaves, β-carotene and lutein, exhibit anti-inflammatory effects by modulating key cytokines and signalling proteins, including TNF, IL-1β, LEP, NF-κB, and COX-2 [77,78]. At the tested extract concentrations, cell viability was maintained, and no morphological alterations were observed, suggesting a genuine moderate anti-inflammatory effect not associated with toxicity.

Due to the limited data on the anti-inflammatory activity of *D. strobilaceus*, evidence from another member of the *Costaceae* family, *Costus* speciosus, is relevant. This species has demonstrated in vitro anti-inflammatory responses mediated by different compounds, namely diosgenin and sesquiterpenes, while targeting similar inflammatory pathways [79,80].

## 5. Conclusions

Beyond its antioxidant and anti-inflammatory properties, *Dimerocostus strobilaceus* represents a previously uncharacterised source of bioactive metabolites with antiproliferative potential. ‘Caña agria’ (*D. strobilaceus*) is a species that stands out for its high concentration of bioactive compounds, which exhibit distinct profiles in the leaves, stems, and seeds. The results showed that the seeds were found to be the main source of phenolic acids, including ferulic (2008.3 mg/100 g DW), caffeic (1325.4 mg/100 g DW) and chlorogenic acids (252.7 mg/100 g DW). The leaves predominantly contained flavonoids such as kaempferol (101.8 mg/100 g DW), quercetin glycosides (54.5 mg/100 g DW), and quercetin (34.7 mg/100 g DW). Leaves exhibited the best antibacterial and antifungal activity (the lower concentration minima inhibitory); however, extracts were inactive against multi-resistant microorganisms. The leaf extract of *D. strobilaceus* demonstrates moderate antiproliferative activity with limited selectivity overall. However, the highest selectivity was observed in hepatocellular carcinoma, highlighting its potential as a source of bioactive compounds while underscoring the need for further fractionation and targeted strategies to enhance tumour-specific effects. In parallel, the extract exhibits moderate anti-inflammatory activity, demonstrating a meaningful suppression of inflammatory responses without cytotoxic effects, which highlights its potential therapeutic relevance and adds to the novel bioactivities reported for this species. These findings suggest that *D. strobilaceus* is a promising source of metabolites with potential applications in antioxidant, antimicrobial, anticarcinogenic and anti-inflammatory therapies, and that more in-depth studies of the mechanisms of action and biotechnological development are warranted.

## Figures and Tables

**Table 1:** Average values of the physicochemical characteristics of ‘Caña agria’ in leaves, stems, and seeds.

Parameters	Leaves	Stems	Seeds
pH	5.5 ± 0.0 ^b^	6.1 ± 0.0 ^a^	4.2 ± 0.1 ^c^
SS (°Brix)	0.1 ± 0.0 ^b^	0.5 ± 0.0 ^a^	0.1 ± 0.0 ^b^
TA (%)	0.2 ± 0.1 ^a^	0.2 ± 0.0 ^a^	0.2 ± 0.0 ^a^
Humidity (%)	80.4 ± 1.5 ^a^	76.8 ± 1.8 ^b^	79.3 ± 0.5 ^a^
Ash (%)	2.7 ± 0.6 ^a^	1.6 ± 0.1 ^c^	1.9 ± 0.2 ^b^
Mineral profile (mg/100 g DW)			
Ca	3559.5 ± 33.9 ^a^	577.2 ± 11.2 ^b^	341.4 ± 13.8 ^c^
Fe	291.5 ± 56.5 ^a^	71.2 ± 0.8 ^b^	42.5 ± 1.1 ^c^
K	4074.8 ± 42.5 ^a^	3288.8 ± 49.5 ^b^	981.3 ± 67.2 ^c^
Mg	390.3 ± 47.9 ^a^	172.7 ± 1.9 ^b^	120.0 ± 2.0 ^c^
Na	14.1 ± 2.0 ^a^	11.7 ± 0.2 ^b^	17.8 ± 3.7 ^a^

Note: Different lowercase letters indicate significant differences between leaves, stems and seeds.

**Table 2:** Phytochemical screening of ‘Caña agria’ in leaves, stems, and seeds.

Metabolites	Leaves	Stems	Seeds
Steroids	-	+	-
Terpenoids	-	+	-
Phenols	+	+	+
Tannins	+	+	+
Alkaloids	-	-	-
Flavonoids	+	-	-
Anthraquinones	-	-	-
Saponins	-	-	-
Acetogenins	+	+	+

Note: -, Negative test result; +, Positive test result.

**Table 3:** Average values of bioactive compounds of ‘Caña agria’ in leaves, stems, and seeds.

Parameters	Leaves	Stems	Seeds
Vitamin C (mg/100 g DW)	16.7 ± 0.7 ^a^	0.4 ± 0.0 ^c^	9.4 ± 0.8 ^b^
Organic acid (mg/100 g DW)			
Citric acid	196.7 ± 19.5 ^a^	117.7 ± 14.0 ^c^	126.2 ± 6.8 ^b^
Malic acid	551.9 ± 30.2 ^b^	615.7 ± 40.0 ^a^	575.9 ± 42.5 ^ab^
Tartaric acid	51.1 ± 0.9 ^b^	76.9 ± 16.3 ^a^	42.7 ± 7.0 ^c^
Total organic acid	799.8 ± 50.6 ^a^	810.3 ± 70.3 ^a^	744.9 ± 28.8 ^b^
Carotenoids (mg/100 g DW)			
Lutein	65.3 ± 4.0 ^a^	1.0 ± 0.0 ^b^	0.4 ± 0.0 ^c^
Zeinoxanthin	2.3 ± 0.0		
Violaxanthin	15.1 ± 2.1 ^a^	0.2 ± 0.0 ^b^	0.1 ± 0.0 ^c^
Zeaxanthin	2.1 ± 0.2 ^a^	0.1 ± 0.0 ^b^	
α-Carotenoid	4.9 ± 0.4		
β-Carotenoid	266.6 ± 5.0 ^a^	1.1 ± 0.1 ^b^	
Carotenoid total	356.4 ± 1.7 ^a^	2.4 ± 0.1 ^b^	0.5 ± 0.0 ^c^
Chlorophylls and their derivatives (mg/100 g DW)			
Chlorophyll b	87.9 ± 7.8 ^a^	6.8 ± 0.1 ^b^	
Pheophytin b	35.4 ± 2.5 ^a^	0.4 ± 0.1 ^c^	1.8 ± 0.1 ^b^
Total chlorophyll	123.3 ± 3.1 ^a^	7.2 ± 0.1 ^b^	2.3 ± 0.0 ^c^
Phenolic compounds (mg/100 g DW)			
Gallic acid	39.8 ± 3.1 ^a^	23.7 ± 2.9 ^b^	8.4 ± 0.1 ^c^
4-Hydroxibenzoic acid	34.9 ± 0.4 ^b^	35.0 ± 1.8 ^b^	44.6 ± 1.1 ^a^
Caffeic acid	27.1 ± 0.8 ^b^	49.8 ± 1.3 ^b^	1325.4 ± 95.3 ^a^
Syringic acid	154.2 ± 1.1 ^a^	86.9 ± 12.2 ^b^	19.1 ± 1.8 ^c^
Chlorogenic acid	35.7 ± 0.0 ^b^		252.7 ± 1.5 ^a^
Ferulic acid	75.9 ± 1.2 ^b^		2008.3 ± 74.1 ^a^
Kaempferol	101.8 ± 1.8		
Quercetin glucoside	54.5 ± 0.0		
Quercetin	34.7 ± 1.5		
Total phenolics	558.7 ± 14.2 ^b^	195.4 ± 20.9 ^c^	3658.5 ± 17.3 ^a^

Note: Different lowercase letters indicate significant differences between leaves, stems and seeds.

**Table 4:** Minimal inhibitory concentration (mg/mL) of ‘Caña agria’ in leaves, stems, and seeds.

	Leaves	Stems	Seeds
Bacteria strain			
*E. coli* ATCC 8739	20.8	10.5	10.5
*P. aeruginosa* ATCC 9027	-	41.8	41.8
*S. aureus* ATCC 6538P	41.7	41.8	41.8
*S. mutans* ATCC 25175	5.2	-	-
Fungi strain			
*C. albicans* ATCC 1031	10.4	41.8	41.8
*C. tropicalis* ATCC 13803	10.4	41.8	41.8
Multidrug-resistant bacteria *			
*Klebsiella pneumoniae*	-	-	-
*Escherichia coli*	-	-	-
*Salmonella enterica serovar Kentucky*	-	-	-
*Enterococcus faecalis*	-	-	-
*Staphylococcus epidermidis*	-	-	-
*Enterococcus faecium*	-	-	-
*Pseudomonas aeruginosa*	-	-	-

Note: - non-active at the tested concentrations; *, These seven clinical multi-drug-resistant strains were provided by the National Health Institute of Ecuador (INSPI).

**Table 5:** Average values of antioxidant activity (mmol TE/100 g DW) of ‘Caña agria’ in leaves, stems, and seeds.

Parameters	Leaves	Stems	Seeds
DPPH	2.8 ± 0.3 ^c^	4.3 ± 0.2 ^b^	5.0 ± 0.2 ^a^
ABTS	4.5 ± 0.5 ^b^	3.4 ± 0.4 ^c^	6.0 ± 0.8 ^a^

Note: Different lowercase letters indicate significant differences between leaves, stems and seeds.

**Table 6:** Half maximal inhibitory concentration values (IC50) (mg/mL) of ‘Caña agria’ leaves against tumour and non-tumour cell lines at 72 h and therapeutic index (TI) values. Values are expressed as mean ± standard deviation, n = 4.

Compound	HeLa		HCT116		THJ29T		HepG2		NIH3T3
	IC_50_	TI ^b^	IC_50_	TI ^b^	IC_50_	TI ^b^	IC_50_	TI ^b^	IC_50_
Caña agria	0.1100 ± 0.00700	1.3	0.1700 ± 0.00800	0.8	0.1000 ± 0.00900	1.4	0.0800 ± 0.01200	1.8	0.1400 ± 0.01200
CDDP	0.0012 ± 0.00001	2.2	0.0022 ± 0.00004	1.2	0.0046 ± 0.00005	0.6	0.0037 ± 0.00004	0.7	0.0027 ± 0.00005

^b^ IC_50_ = (NIH3T3)/IC_50_ (tumour cell).

## Data Availability

The original contributions presented in this study are included in the article. Further inquiries can be directed to the corresponding author.
